# Role of Sostdc1 in skeletal biology and cancer

**DOI:** 10.3389/fphys.2022.1029646

**Published:** 2022-10-21

**Authors:** Xiaoyang Tong, Chenyu Zhu, Lifei Liu, Mei Huang, Jiake Xu, Xi Chen, Jun Zou

**Affiliations:** ^1^ School of Exercise and Health, Shanghai University of Sport, Shanghai, China; ^2^ Department of Rehabilitation, The People’s Hospital of Liaoning Province, Shenyang, China; ^3^ School of Biomedical Sciences, University of Western Australia, Perth, WA, Australia; ^4^ School of Sports Science, Wenzhou Medical University, Wenzhou, China

**Keywords:** SOSTDC1, skeletal biology, cancer, Wnt, BMP

## Abstract

Sclerostin domain-containing protein-1 (Sostdc1) is a member of the sclerostin family and encodes a secreted 28–32 kDa protein with a cystine knot-like domain and two N-linked glycosylation sites. Sostdc1 functions as an antagonist to bone morphogenetic protein (BMP), mediating BMP signaling. It also interacts with LRP6, mediating LRP6 and Wnt signaling, thus regulating cellular proliferation, differentiation, and programmed cell death. Sostdc1 plays various roles in the skin, intestines, brain, lungs, kidneys, and vasculature. Deletion of Sostdc1 gene in mice resulted in supernumerary teeth and improved the loss of renal function in Alport syndrome. In the skeletal system, Sostdc1 is essential for bone metabolism, bone density maintenance, and fracture healing. Recently, Sostdc1 has been found to be closely related to the development and progression of multiple cancer types, including breast, renal, gastric, and thyroid cancers. This article summarises the role of Sostdc1 in skeletal biology and related cancers to provide a theoretical basis for the treatment of related diseases.

## Introduction

Sclerostin domain-containing protein-1 (Sostdc1), also named as uterine sensitisation-associated gene one protein (Usag-1), CDA019, Wise, ectodermal BMP inhibitor (Ectodin), and Sclerostin-like protein (Sostl), is highly expressed in the skin, intestines, brain, skeletal muscles, lungs, kidneys, and vasculature (Millan et al., 2019). Furthermore, it is expressed in the bone periosteum and mesenchymal stem cells, where it supports bone formation and remodelling (Liu et al., 2008). Moreover, the role of Sostdc1 in skeletal development is related to its high degree of homology with sclerostin (SOST); together, they form a novel sub-group of cysteine knot proteins (Itasaki et al., 2003; Semenov et al., 2005; Ellies et al., 2006). Sostdc1 has been widely studied in the development of tooth, bone fracture, cancers, kidney disease, vasculature, and hair follicle formation (Collette et al., 2013; Togo et al., 2016; Xiao et al., 2018; Chicana et al., 2019; Millan et al., 2019; Schupp et al., 2021). Deletion of the Sostdc1 gene in mice resulted in supernumerary teeth (Murashima-Suginami et al., 2008), and improved the loss of renal function in Alport syndrome (Tanaka et al., 2010). In this review, we summarise the role of Sostdc1 in the skeletal system and the development of cancers, providing novel insights into its potential mechanisms of action.

### Molecular structure, expression and function of Sostdc1

**FIGURE 1:**
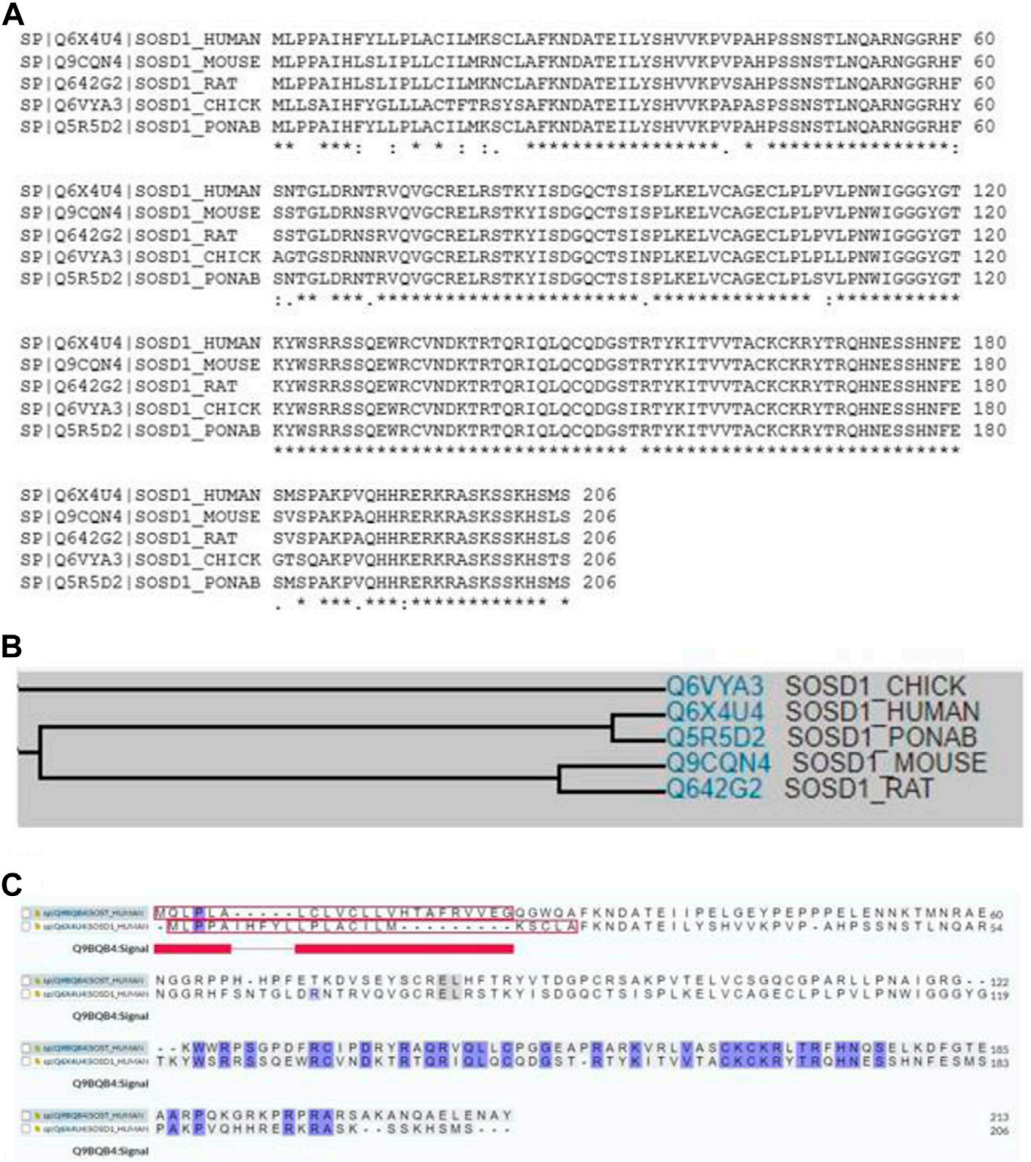
**(A)** Multiple sequence alignment showing substantial sequence homology among Sostdc1 amino acid sequences in human, mouse, rat, chick, and ponab. **(B)**Family tree analyses among Sostdc1 amino acid sequences in chick, human, ponab, mouse, and rat. **(C)** Sequence comparison between human SOST and Sostdc1.

Sostdc1 is a 28–30 kDa secretory protein that behaves like a monomer, with no extra cysteine residues present in Noggin; meanwhile, DAN (a BMP antagonist that inhibits neoplastic transformation) is required to create intermolecular disulfide bridges (Laurikkala et al., 2003; Avsian-Kretchmer and Hsueh, 2004; Yanagita et al., 2004). The Sostdc1 mRNA encodes a 206-amino acid long putative protein that contains a C-terminal cystine knot-like motif, two N-linked glycosylation sites and a predicted N-terminal secretion signal (Simmons and Kennedy, 2002). The amino acids show a high degree of sequence conservation, with the cDNA sequences in mouse and rat being 98% and 97% identical to that in humans, respectively (Yanagita, 2005). Multiple sequence alignment analysis shows a high degree of sequence similarity among homologs of Sostdc1, including human, mouse, rat, chick, and ponab homologues (Figure 1A). Family tree analysis shows that human Sostdc1 is most closely related to ponab homologues, followed by rat, mouse, and chick. (Figure 1B). Furthermore, the sequence comparison between human SOST (the protein of the SOST gene) and human Sostdc1 as showed in Figure 1C, demonstrated that Sostdc1 has 38% amino acid identity with SOST (Kusu et al., 2003). SOST shows higher specificity for the Lrp4 co-receptor (Ohazama et al., 2008), whereas Sost preferentially binds to the Lrp5 and Lrp6 co-receptors (Collette et al., 2010; van Dinther et al., 2013).

## Role of Sostdc1 in skeletal biology

Accumulating evidence suggests that Sostdc1 plays an essential role in bone metabolism, bone density maintenance, and fracture healing (Ellies et al., 2014; Collette et al., 2016; Sun et al., 2019).

### Sostdc1 negatively regulates bone formation

**FIGURE 2:**
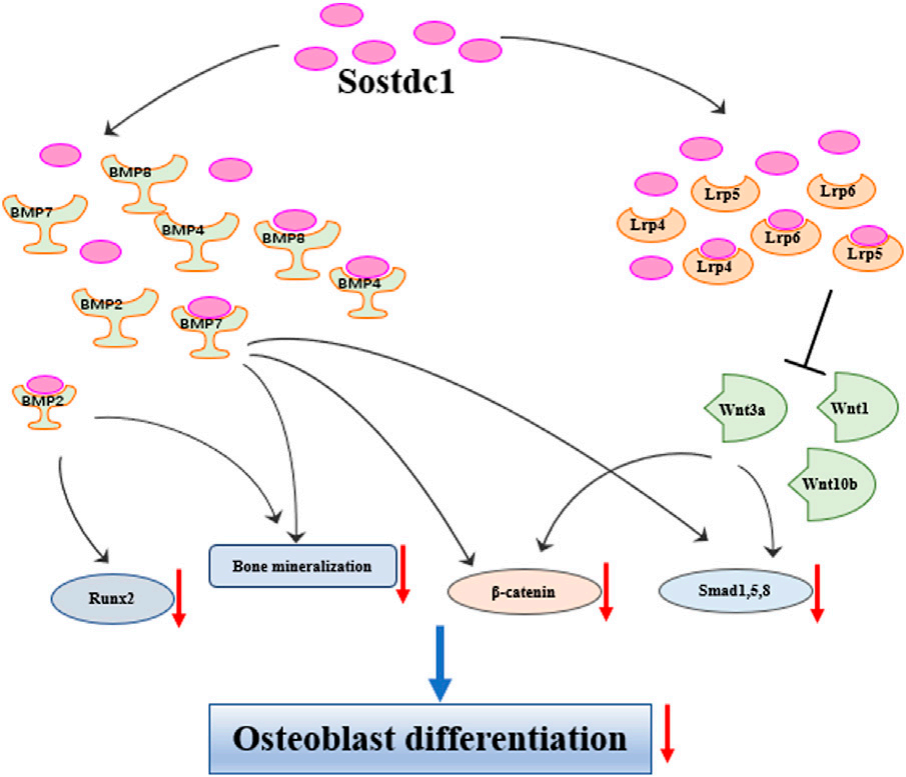
Sostdc1 negatively regulates bone formation through Wnt and BMP pathways.

Under healthy conditions, bone homeostasis requires the balance between bone formation and resorption (Kular et al., 2012). Sostdc1 regulates bone formation by affecting osteoblast differentiation. Oestrogen treatment in postmenopausal women decreased the levels of two key inhibitors of Wnt/BMP signalling in the bone, Sost and Sostdc1, and reduced NF-κB signalling (Fujita et al., 2014). A study from Genome-wide linkage analysis revealed that quantitative trait loci on chromosome 7p21.1 was essential for femoral neck bone mineral density (BMD) in Chinese families (LOD = 3.68), while Sostdc1 is located on 7p21.1. The study also correlated that a polymorphism in Sostdc1 was also linked to low lumbar BMD, but not to femoral neck or total hip BMD in women (He et al., 2011), indicating that Sostdc1 genetic variants have an impact on Chinese women’s ability to achieve and maintain their maximum bone mass. This association analysis in humans implies that mutations affecting Sostdc1 function can adversely affect trabecular BMD more than cortical BMD since the lumbar vertebrae are predominantly made of trabecular bone (He et al., 2011). Nicole et al. found that Sostdc1 knockout mice (Sostdc1^−/−^) had high cortical bone levels in the femur, suggesting that Sostdc1 had different effects on trabecular and cortical bone (Collette et al., 2016). This was attributed to the fact that the absence of Sostdc1 locally promotes osteoblast activity in the periosteum, leading to larger and thicker bone cortices. Ellies et al. used genetic approaches to show a transient increase in BMD and an Lrp5-dependent increase in osteoblast proliferation rate in Sostdc1^−/−^ mutant mice. Interestingly, they also found that Sostdc1 was required to potentiate chondrocyte proliferation, likely serving as a positive modulator of Wnt activity (Ellies et al., 2014). Faraahi et al. (2019) evaluated the function of Sostdc1 using *in vitro* osteoblast differentiation assays and demonstrated that the calcium mineralisation induced by BMP2 and BMP7, as well as Runx2 gene expression induced by BMP2, in OB progenitor differentiation was inhibited in the presence of Sostdc1. In addition, Sostdc1 significantly reduced β-catenin levels and phosphorylated Smads 1, 5, and 8 protein levels induced by Wnt3a, and β-catenin expression induced by BMP7, indicating that Sostdc1 antagonises Wnt-BMP signalling crosstalk in osteoblast progenitors. Interestingly, Sostdc1 is also positively regulated by Hedgehog (HH) signalling, accompanied by the downregulation of key regulators of BMP signalling effectors and osteogenesis, and ectopic HH-Smo signalling downregulates the Wnt/BMP pathways, at least in part *via* the upregulating Sostdc1, leading to cleft palate and defective osteogenesis (Hammond et al., 2018). Overall, Sostdc1 negatively affects osteoblast differentiation mediated by the Wnt/BMP signalling pathways (Figure 2).

### Sostdc1 negatively regulates fracture rehabilitation

As indicated earlier, Sostdc1 is expressed in the periosteum, which negatively regulates bone formation and fracture healing. A study investigating the regulatoryrole of Sostdc1 in fracture repair found that Sostdc1^−/−^ mice with mid-diaphyseal femoral fractures showed a more rapid cartilage turnover, with a significantly thicker cortical shell and remodelled bone 21 days post fracture, and that Sostdc1-positive cells participate in early fracture repair. At later time points of the repair process, Sostdc1^−/−^ mice showed enhanced mineral density, callus volume, and BV, suggesting that osteoblast differentiation increased early in the repair process, translating to greater bone formation. In addition, the growing in the metaphysis and activated β-catenin levels in cortical bone reveal that Sostdc1 affects Wnt/β-catenin signalling, implying that the increase in cortical bone was caused by elevated Wnt signalling (Collette et al., 2016). Additionally, Sostdc1 plays a significant role in the regulation of nonunions after a fracture. Sun et al. (2019) revealed that Sostdc1 expression was downregulated by increasing miR-26a expression in a nonunion rat model by removing the periosteum; miR-26a promoted osteogenic differentiation of MSCs *in vitro* and fracture healing in rats with nonunions *in vivo*, likely by targeting Sostdc1 and Sostdc1-mediated Wnt/β-catenin signalling pathway.

Collectively, these results reveal that Sostdc1 may play an essential role in bone metabolism. As an antagonist of the Wnt and BMP signalling pathways, Sostdc1 may be a key regulatory protein and potential therapeutic agent for bone diseases.

### Sostdc1 regulates tooth development

Sostdc1 is also involved in tooth development, including the determination of tooth size, number, and cusp pattern (Arikan et al., 2018). Kassai et al. showed that Sostdc1-deficient mice have highly altered cusp patterns, enlarged enamel knots, and extra teeth, suggesting that Sostdc1 is vitally important for the development of teeth (Kassai et al., 2005). Blocking Sostdc1 function by knocking out Sostdc1 or administering anti—Sostdc1 antibodies decreased congenital tooth agenesis as a result of various genetic abnormalities in mice (Murashima-Suginami et al., 2021). Munne et al. (2009) demonstrated that Sostdc1 expression is restricted to the mesenchyme and as a result of Wnt signalling activation, the supernumerary tooth induction may be limited by the dental mesenchyme. Furthermore, Sostdc1 expression is localised to the mesenchyme and epithelium of the rudimentary maxillary incisor tooth organ, while Sostdc1 takes control of the number of teeth in the maxillary incisor area by regulating apoptosis (Murashima-Suginami et al., 2007). Enhanced Wnt- and BMP-mediated signal transduction in Sostdc1^−/−^ mice’s dental mesenchyme may increase cell proliferative ability in the dental mesenchyme at the early bell stages or cap, leading to increased supernumerary tooth formation and tooth size (Saito et al., 2016). In addition, Ahn et al. (2010) provided evidence that Sostdc1 suppressed incisor vestigial buds or the survival of diastema during tooth development and inactivation of Sostdc1-activated Wnt signalling, resulting in vestigial tooth buds in the normally toothless diastema area and displaying increased continuous development and proliferation; this leading to supernumerary teeth. The FGF and SHH pathways are major targets of the downstream of Wnt/β-catenin signalling regulated by Sostdc1. A subsequent report demonstrated that BMP7 induces supernumerary tooth formation. However, inducing the formation of extra teeth using BMP7 alone is impossible. The interaction between BMP7 and Sostdc1 genes regulates supernumerary maxillary incisor formation (Kiso et al., 2014). Therefore, the mechanism to inhibit deciduous incisors in mice involves the expression of Sostdc1, which inhibits BMP7 signalling, resulting in degeneration and apoptosis of rudimentary tooth germs (Kiso et al., 2014).

### Sostdc1 is an antagonist of both Wnt and BMP signalling pathways

Sostdc1 controls tooth number and morphology by inhibiting the Wnt and BMP signalling pathways (Arikan et al., 2018). Sostdc1 is regarded as an antagonist of both the Wnt and BMP signalling pathways (Chao et al., 2018; Millan et al., 2019; Gipson et al., 2020; Wu et al., 2020). The Wnt signalling pathway plays an important role in regulating bone cell differentiation, proliferation, growth, survival, development, regeneration, self-renewal, and fate determination (Miller, 2002; Marini et al., 2022). Activation of Wnt/β-catenin signalling initially from the Wnt protein binds to frizzled receptor and Lrp5/6 co-receptors (Hay et al., 2005; Huybrechts et al., 2020). The Wnt signalling pathway plays various roles in the skeletal development, for instance, the genesis of osteoblasts, osteoclasts, chondrocytes and so on (Krishnan et al., 2006; Kular et al., 2017). Thus, the Wnt signalling pathway directly and indirectly controls both osteoclastogenesis and osteoblastogenesis. Consequently, maintaining appropriate levels of Wnt activity is essential for bone homeostasis. Reporter assays have established that Sostdc1 blocks Wnt1, Wnt3a, and Wnt10b activities, inhibiting Wnt signalling (Yanagita et al., 2004; Beaudoin et al., 2005; Blish et al., 2008). Furthermore, Sostdc1 inhibits Wnt signalling by binding to the extracellular domain of Wnt co-receptors Lrp4, Lrp5, and Lrp6 (Laurikkala et al., 2003; Ohazama et al., 2008; Ahn et al., 2010). Interestingly, Sostdc1 was found to compete binding to Lrp6 with Wnt8, suggesting a mechanism for inhibiting the Wnt pathway that involves Sostdc1 blocking the binding of ligands and receptors (Itasaki et al., 2003). Simultaneously, Shh signalling occurs upstream of Sostdc1, suggesting the negative feedback loop of Wnt-Shh-Sostdc1 is a pivotal mechanism modulating the spatial patterning of teeth, with Wnt, Shh, and Sostdc1 acting as activators, mediators, and inhibitors, in reaction-diffusion models (Cho et al., 2011). As Sostdc1 and Shh inhibit cusp pattern formation, suppressing Shh and Sostdc1 may result in the inhibition zone at each cusp, decreasing the intercuspal distance. Shh inhibits cusp pattern formation by modulating Wnt signalling through positive regulation of Sostdc1 (Kim et al., 2019). Latest research shows that diphyodontic dentition formation may be associated with the Sostdc1-Wnt negative feedback loop and FGF signal upregulation (Mao et al., 2022). Modulation of Wnt signalling by Sostdc1 and Dkk2 plays a pivotal role in the odontogenic pathway dependent by Msx1 in early tooth morphogenesis (Lee et al., 2022). Moreover, cementum apposition is modulated by HH signalling negatively in a Wnt/β-catenin/Osx-dependent manner. Dkk1 and Sostdc1 are regulated by Smo activation and play crucial roles in the reduction of β-catenin activity and Osx expression (Choi et al., 2020).

Sostdc1 is not only an antagonist of the Wnt signalling pathway, but also an antagonist of the BMP signalling pathway. BMPs were originally determined as proteins that induce cartilage formation and ectopic bone *in vivo* (Salazar et al., 2016; Hashimoto et al., 2020). BMPs are signalling molecules that stimulate osteoblast differentiation (Hogan, 1996; Salazar et al., 2016), and in mesenchymal stem cells, the critical transcription factors Runx2 and osterix are induced by BMP2 and BMP7, which promote osteoblast differentiation (Lee et al., 2003a; Lee et al., 2003b; Zhang et al., 2020; Zhang et al., 2021). Runx2 and Sostdc1 function antagonistically during tooth formation, and Runx2 inhibits the Wnt and/or BMP signalling pathways regulated by Sostdc1, and Runx2 expression is induced by BMP signalling independent of Sostdc1 (Togo et al., 2016). Moreover, Sostdc1 binds to Lrp6 through one of the three loops formed by the cysteine knot, and Sostdc1 deletion constructs lacking the Lrp6-interacting loop domain bind to BMP4 and inhibit BMP signals (Lintern et al., 2009). Interestingly, functional assays also revealed that the ability of Sostdc1 to block Wnt1 activity is prevented by BMP4 through Lrp6, whereas the ability of Sostdc1 to suppress BMP4 is prevented by excess Lrp6, implying a preference for Sostdc1 over BMP4 in Lrp6 binding (Lintern et al., 2009). Sostdc1 also suppresses the phosphorylation of R-Smads-1, -5, and -8 induced by BMP7, and Wnt-3a signalling (Blish et al., 2008). Sostdc1 suppresses BMP signalling through binding to BMP *via* its cystine knot structural motif (Lintern et al., 2009; Cruciat and Niehrs, 2013). The affinity of Sostdc1 for the Lrp6 receptor protein was one-fold higher than the affinity of Sostdc1 for the BMP2 ligand protein, while the Sostdc1 protein exhibited a three-fold higher affinity for binding BMP7 in comparison with Lrp6 (Faraahi et al., 2019). Sostdc1 also inhibits BMP-2, -4, -6, and BMP7 activity in mouse preosteoblast MC3T3-E1 cells and binds to these BMPs with high affinity (Laurikkala et al., 2003).

Taken together, Sostdc1 plays a significant role in regulating tooth development, including tooth size, number, and cusp pattern. Moreover, the regulation of Sostdc1 involves the Wnt and BMP signalling pathways, and the development of potential tooth germs.

## Role of Sostdc1 in cancer

Sostdc1 is involved in the progression and development of multiple cancer types, including breast, gastric, renal, bladder, and thyroid cancers (Blish et al., 2008; Gopal et al., 2013; Rawat et al., 2014; Li et al., 2020). Sostdc1 deficiency is associated with larger tumour sizes (Clausen et al., 2011). Notably, Sostdc1 modulates NK cell maturation and Ly49 receptor expression in a cell-extrinsic manner from both haematopoietic and nonhematopoietic sources. NK cells are innate-like lymphocytes that eliminate virus-infected and cancerous cells, suggesting that Sostdc1 plays an important regulatory role in related cancers (Millan et al., 2019).

### Sostdc1 methylation inhibits cancer development

Sostdc1 can exert inhibitory effects on cancer cells (Kim et al., 2022). Recent studies have highlighted that epigenetic modifications result in Sostdc1 downregulation in cancer (Blish et al., 2010; Tesfay et al., 2015). In addition, Sostdc1 methylation suppresses its expression and activity (Gopal et al., 2013; Xiao et al., 2018). A previous study demonstrated that Sostdc1 inhibits hepcidin secretion and suppresses the proliferation of thyroid cancer cells (Zhou et al., 2018). In addition, Sostdc1 expression was downregulated in thyroid cancer, which was associated with the hypermethylation of its promoter. E4BP4 inhibited Sostdc1 through recruiting G9a to its promoter, methylating the Sostdc1 promoter, and suppressing its expression. However, the underlying mechanisms have yet to be fully elucidated (Zhou et al., 2018). In prostate tumours, the Sostdc1 promoter was hypermethylated in prostate tumours, leading to the suppression of hepcidin secretion due to attenuation of BMP4/7-mediated Smad phosphorylation, resulting in inhibition of cell survival (Lintern et al., 2009). Sostdc1 was also frequently downregulated in primary breast cancers (98.2%), and Sostdc1 promoter hypermethylation at CpG sites inhibited cell proliferation and survival, while E4BP4 reduced Sostdc1 expression and its promoter activity in breast cancer cells (Rawat et al., 2014). Sostdc1 is the only gene associated with survival in patients with metastatic breast cancer (Clausen et al., 2011; Rawat et al., 2014), indicating that Sostdc1 plays multiple roles in metastatic breast cancer. In addition, the regulation of breast cancer by Sostdc1 is correlated with its methylation, as the methylation levels of Sostdc1 were evidently lower in control tissues than in cancer tissues. Consistently, the methylation level is associated with breast cancer prognosis and hence, can be used as a diagnostic marker (Xiao et al., 2018). Moreover, multivariate diagnostic analysis of the methylation status of WIF1, Sostdc1, and DACT2 showed up to 91% specificity and 100% sensitivity in discriminating breast cancer (invasive and non-invasive) from benign tumours and controls and may be a complementary tool for breast cancer diagnosis (Rajkumar et al., 2022). A gastric cancer study found that Sostdc1 expression is modulated by epigenetic mechanisms and its downregulation may be caused by promoter hypermethylation (a potential prognostic factor) and tumour suppression, with the expression of Sostdc1 suppressing cell proliferation and cell cycle progression (Gopal et al., 2013). Further research is required to provide a comprehensive understanding of the role of Sostdc1 as a prognostic marker in gastric cancer as well as its tumour suppressor properties. Consistent with the potential role of Sostdc1 as a tumour suppressor, Blish et al. (2010) demonstrated that Sostdc1 expression was significantly lower in both paediatric Wilms tumours and adult clear cell renal carcinoma. They also suggested that alternative mechanisms, for example, epigenetic silencing of Sostdc1, which may be an essential factor for the reduction of Sostdc1 protein and mRNA levels in renal cancer. Notably, Sostdc1 expression was upregulated in normal kidneys but downregulated in renal cancer (Blish et al., 2008; Xu et al., 2022).

### Sostdc1 inhibits BMP signalling pathways

Sostdc1 regulates the development of cancers through BMP signalling pathways. For example, a study by Zhou et al. (2017) showed that Sostdc1 compromised the migratory and invasive properties and epithelial-mesenchymal transition (EMT) activity of follicular thyroid cancer cells by suppressing the activities of the PI3K/Akt and MAPK/ERK signalling pathways. Furthermore, Sostdc1 showed tumour suppressor properties in gastric cancer, and its silencing enhanced the movement of cancer cells, accelerated tumour growth, and promoted the formation of lung metastases by inhibiting the SMAD and c-Jun N signalling pathways (Cui et al., 2019). In addition to BMP4 overexpression of in diffuse-type gastric cancer cells, BMP signalling was modulated by secreted-type antagonists such as Sostdc1, CER1, CKTSF1B1, Noggin, and Chordin (Katoh and Katoh, 2004).

Interestingly, Clausen et al. (2011) showed that Sostdc1 protein and mRNA levels decreased in breast cancer cells and high Sostdc1 mRNA levels were correlated with increased distant metastasis-free survival in patients with breast cancer. Sostdc1 blocks the Smad phosphorylation induced by BMP7 without diminishing BMP2 or Wnt3a-induced signalling in breast cancer cells, indicating that Sostdc1 is a clinically important extracellular regulator of various signalling pathways in breast cancer.

Furthermore, Sostdc1 promoted invasion and liver metastasis by inducing ALCAM-mediated PI3K/AKT and Src activation and overcoming BMP4-specific antimetastatic signals in colorectal cancer (Bartolome et al., 2020). Similarly, Sostdc1 inhibited the migration, proliferation, and invasion of epithelial ovarian cancer (EOC) cells, while knocking down Sostdc1 rescued the inhibition of si-lncRNA CDKN2A-AS1 on those processes. These results demonstrate that CDKN2A-AS1 activates the BMP-SMAD signalling pathway by directly binding to Sostdc1 and promoting EOC tumour growth. Targeting the CDKN2A-AS1/Sostdc1 axis may be a novel therapeutic strategy to treat EOC (Zhao et al., 2021). Additionally, Sostdc1 levels were lower in renal clear cell carcinoma, and Sostdc1 suppressed renal carcinoma cell proliferation by inhibiting Wnt3a signalling and the phosphorylation of R-Smads-1, -5, and -8 induced by BMP7. The restoration of Sostdc1 signalling may suggest a novel strategy for treating renal clear cell carcinoma (Blish et al., 2008). However, interestingly, a previous systematic review concluded that Sostdc1 inhibits the progression of kidney-related cancers but promotes some kidney diseases (Li et al., 2021), including acute and chronic renal injuries (Tanaka et al., 2008), and renal fibrosis (Hamasaki et al., 2012).

### Other pathways that regulate cancer

Sostdc1 also inhibits non-small cell lung cancer (NSCLC) (Herbst et al., 2018), Liu et al. (2016) showed that Sostdc1 expression was significantly downregulated in NSCLC Additionally, patients with higher Sostdc1 expression had significantly better prognoses compared to patients with lower Sostdc1 expression. Another study revealed that Sostdc1 overexpression inhibited NSCLC cell proliferation, migration, and invasion, along with the osteoclastogenesis induced by cancer cells, whereas Sostdc1 knockdown had the opposite effect. In NSCLC bone metastatic lesions, Sostdc1 was downregulated compared to primary tumours, and low Sostdc1 expression predicted a poor NSCLC prognosis (Chen et al., 2018). In Wilms tumour, Sostdc1 has been identified as the likely tumour suppressor gene located in the chromosome 7p21 region that is lost following homozygous deletion, and the loss of Sostdc1 may augment signals in the Wnt pathway, which may be a prime candidate for 7p tumour suppressor genes, and may play an important role in Wilms tumourigenesis progression (Ohshima et al., 2009). In addition, exosomal circ_0001190 overexpression suppressed cell vitality, migration, proliferation, and invasion of gastric cancer through the miR-586/Sostdc1 axis. Circ _0001190 served as an miR-586 sponge, regulating Sostdc1 expression, and miR-586 promoted gastric cancer advancement by interfering with Sostdc1 (Liu et al., 2022). Sostdc1 also increased the cell apoptotic rate and inhibited the proliferative ability in acute myeloid leukaemia (AML) by suppressing the Wnt/β-catenin pathway. Sostdc1 levels in the bone marrow of patients with AML were lower than those in healthy individuals, and patients with low levels of Sostdc1 had shorter survival times (Li et al., 2022). Furthermore, in the 5T2MM murine model of multiple myeloma (MM), Sostdc1 was shown to be expressed in low levels in MM and OB lineage cells while these cells were separately cultured, but its expression was substantially also induced in cell types when they were co-cultured, suggesting that the expression of Sostdc1 in 5TGM1-infiltrated bones could suppress osteoblast differentiation in bone and tumor microenvironment (Faraahi et al., 2019).

Overall, Sostdc1 downregulation was associated with poor prognosis and tumour aggressiveness in several types of cancer (Table 1). Sostdc1 plays an inhibitory role in tumourigenesis, and its downregulation enhances cancer cell proliferation, colony formation, and tumour expansion. However, the potential role of Sostdc1 as a therapeutic target in related cancers requires further investigation.

**TABLE 1:** Role of Sostdc1 in cancer.

Cancer types	Target	Biological function	References
Prostate tumors	Promoter hypermethylation BMP4/7-Smad	Inhibit cell survival	Tesfay et al. (2015)
Thyroid cancer	Promoter hypermethylation E4BP4/G9a/Sostdc1/hepcidin	Inhibit cell proliferation	Zhou et al. (2018)
Breast cancer	Promoter hypermethylation E4BP4/Sostdc1	Inhibit cell proliferation and survival	Rawat et al. (2014)
Gastric cancer	Promoter hypermethylation	Inhibit cell proliferation and cell cycle progression	Gopal et al. (2013)
Follicular thyroid cancer	PI3K/Akt, MAPK/ERK	Inhibit cell proliferation, migration and EMT	Zhou et al. (2017)
Gastric cancer	SMAD, c-Jun N	Inhibit tumorigenesis and lung metastases	Cui et al. (2019)
Breast cancer	BMP7-Smad, BMP2,Wnt3a	Inhibit tumorigenesis	Clausen et al. (2011)
Epithelial ovarian cancer	lncRNA CDKN2A-AS1/BMP-SMAD	Inhibit cell proliferation, migration, and invasion	Zhao et al. (2021)
Renal clear cell carcinoma	BMP7-Smad1,5,8, Wnt3a	Inhibit cell proliferation	Blish et al. (2008)
Non-small cell lung cancer	p21Cip/p27Kip-Rb-E2F	Inhibit cell proliferation, migration, invasion, and cancer cell-induced osteoclastogenesis	Liu et al. (2016); Chen et al. (2018)
Wilms tumor	Wnt	Inhibit tumorigenesis	Ohshima et al. (2009)
Gastric cancer	Exosomal circ_0001190- miR-586/Sostdc1	Inhibit cell vitality, proliferation, migration, and invasion	Liu et al. (2022)
Acute myeloid leukemia	Wnt/β-catenin	Inhibit cell proliferation and increase the apoptotic rate of cells	Li et al. (2022)
Colorectal cancer	BMP4- ALCAM- Src, PI3K/AKT	Promote invasion and liver metastasis	Bartolome et al. (2020)

## Summary

Sostdc1, a regulator of the BMP and Wnt signalling pathways, is involved in the development and progression of several diseases. We have summarised the effects of Sostdc1 on bone metabolism, bone density maintenance, and fracture healing. Sostdc1 has a negative regulatory effect on bone metabolism and inhibits fracture healing through Wnt and BMP signalling pathways (Figure 2). As a modulator of cell proliferation and differentiation, Sostdc1 is associated with the development and progression of multiple cancer types, including breast, renal, gastric, and thyroid cancers. Sostdc1 inhibits the proliferation of related cancer cells through its methylation, BMP signalling pathways, and other mechanisms. In summary, Sostdc1 plays a crucial regulatory role in several diseases. Further studies are required to fully define the underlying mechanism of action and its therapeutic potential.
